# Multiplex PCR approach to simultaneously identify several mutations in fine needle cytology thyroid samples

**DOI:** 10.18632/oncotarget.17656

**Published:** 2017-05-07

**Authors:** Emilia Vuttariello, Marco Borra, Elvira Mauriello, Celeste Calise, Barbara D'Andrea, Anna Capiluongo, Franco Fulciniti, Anna Cipolletta, Mario Monaco, Luciano Pezzullo, Gennaro Chiappetta

**Affiliations:** ^1^ Functional Genomics Unit, Istituto Nazionale Tumori, IRCCS, Fondazione G. Pascale, Naples, Italy; ^2^ Molecular Biology and Bioinformatics Unit, Stazione Zoologica “A.Dorhn”, Naples, Italy; ^3^ CMO, Naples, Italy; ^4^ Clinical Cytopathology Service, Institute of Pathology, Locarno, Switzerland; ^5^ SC Anatomia Patologica e Citopatologia, Istituto Nazionale Tumori, IRCCS, Fondazione G. Pascale, Naples, Italy; ^6^ Thyroid Surgery Unit, Istituto Nazionale Tumori, IRCCS, Fondazione G. Pascale, Naples, Italy

**Keywords:** multiplex PCR, thyroid, fine needle cytology, genetic testing, Sanger sequencing

## Abstract

The most frequent initial manifestation of thyroid cancer is the appearance of a nodule. More than 20% of the general population has a palpable thyroid nodule and the percentage rises to 70% based on ultrasound identification. In 95% of cases the nodule is simply a hyperplastic or benign lesion. The most reliable diagnostic test for thyroid nodules is fine needle aspiration (FNA), but cytological discrimination between malignant and benign follicular neoplasms remains difficult. Cytological analysis is now, almost routinely, being combined with molecular genetics to enable the pathologist to make a more objective diagnosis. In this study, we performed the molecular analysis using a new simplified procedure that involves a panel of *BRAF*, *RAS*, *RET* and *RET/PTC* gene mutations in easily obtainable FNA samples, in the attempt to improve the efficacy of the FNA diagnosis of thyroid nodules and thus patient management. In this new procedure, PCR and sequencing analysis are used to detect point mutations, and, in parallel, RT-PCR is used to detect the chimeric RET/PTC1 and RET/PTC3 transcripts in RNA extracted from FNA.

## INTRODUCTION

Thyroid cancer is the most common malignant tumor of the endocrine system [1]. Most thyroid cancers derive from thyroid follicular cells that give rise to well-differentiated papillary (PTC) and follicular (FTC) carcinomas, and undifferentiated forms such as anaplastic carcinoma (ATC) [2], while medullary thyroid carcinomas (MTC) derive from thyroid parafollicular cells or C cells. Medullary thyroid carcinoma accounts for approximately 5–10% of all thyroid cancers, 75% are sporadic and 25% hereditary. A nodule is usually the initial manifestation of thyroid cancers but in 95% of cases it is simply a hyperplasic or benign lesion. Therefore, given the low mortality rate of thyroid cancer and the low percentage of malignant thyroid nodules, an accurate diagnosis of lesions will spare patients unnecessary surgery.

Although thyroid nodules are mostly diagnosed by fine needle aspiration (FNA), the cytological discrimination between malignant and benign follicular neoplasms remains difficult despite their distinctive cytomorphological features, PTC, MTC and ATC are easily diagnosed. However, FNA may yield insufficient material or a low number of tumor cells, and many thyroid FNAs are “indeterminate”. In the latter case, FNA sampling is usually repeated or in some cases, surgery is performed in the attempt to establish a diagnosis. These procedures result in additional morbidity and higher health care costs. Moreover, patients with malignant tumors and indeterminate FNA cytology typically undergo limited surgery, *i.e*. lobectomy, and once malignancy is established by pathological examination of the excised nodule, these patients must undergo a second operation to complete the thyroidectomy, which also results in additional morbidity and costs. Moreover, 1–3% of nodules diagnosed as benign on FNAs are later found to be malignant on follow-up [3]; the consequent delay in treatment places patients at risk of disease progression.

Molecular testing of FNA samples significantly improves the accuracy of the cytological diagnosis of thyroid nodules. Therefore, procedures to improve the sensitivity and specificity of FNA diagnosis could have a significant impact on clinical care [4–6].

A number of genetic mutations are associated with thyroid cancer. The most common genetic mutations in PTC are point mutations involving the *BRAF* and *RAS* genes, while the most common chromosomal rearrangement is *RET/PTC*, which is involved in the mitogen-activated protein kinase (MAPK) pathway [7–9]. These mutations are found in more than 70% of PTCs, and are mutually exclusive [10–12]. *RAS* mutations or the *PAX8/PPARγ* rearrangement, which are mutually exclusive, occur in about 80% of FTCs [13]. Medullary thyroid carcinomas frequently feature point mutations in the RET gene (both hereditary and sporadic). In fact, MTC is transmitted in an autosomal-dominant pattern [14] in multiple endocrine neoplasia (MEN) syndrome (MEN 2A and MEN 2B) and in familial medullary thyroid carcinoma (FMTC). Genetic screening should be performed even in apparently sporadic cases, because about 5% of sporadic cases of MTC are hereditary [15]. Many other genetic alterations, namely, *PIK3CA, PTEN, b-Catenin (CTNNB1), TP53, AKT*, and *TRK* rearrangements are involved in thyroid cancer but are rare.

According to the American Thyroid Association guidelines [16], molecular markers such as *BRAF, RAS* and *RET/PTC* may be used to type patients with indeterminate FNA cytology and thus help guide management. The classical methods used to detect mutations in these more informative genes involve the use of individual amplifications of exon 15 of *BRAF*, exons 2 and 3 of *H-K-NRAS* and *RET/PTC1* and *RET/PTC3* rearrangement, each analyzed at a different annealing temperature followed by forward and reverse sequencing. We have developed two multiplex PCR assays that enable the simultaneous identification of 15 point mutations. The first PCR assay involves the use of primers to amplify the most important exons of the *RET* gene. The second consists in the amplification of the *BRAF*, *H-K* and *NRAS* gene mutated regions.

## RESULTS AND DISCUSSION

**Figure 1:**
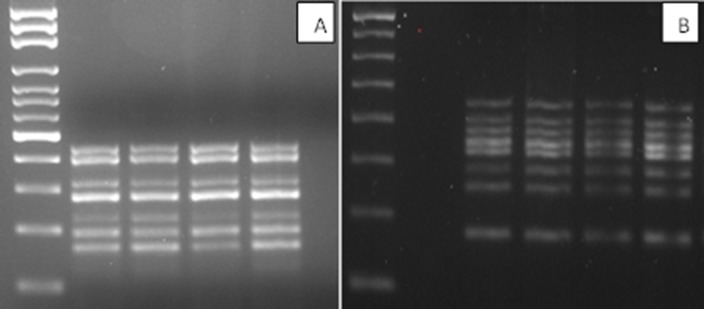
(**A**) BRAF-RAS multiplex amplification gel (**B**) RET multiplex amplification gel (2% agarose gel in 1X TBE).

We developed an efficient and inexpensive method of nucleic acids extraction that yields sufficient material to amplify simultaneously a gene panel to characterize thyroid tissue using separate DNA and RNA extraction kits. A kit is also available for the simultaneous extraction of nucleic acids. The nucleic acid concentrations differed greatly: an average of 22 ng/μl (total 4400 ng/μl) in the case of DNA, and an average of 61 ng/μl (total 3660 ng/μl) in the case of RNA, depending on the type of sample, and also on the different characteristics of the nodule such as the presence of calcifications or necrosis that makes the really poor material. It should be noted that we also successfully amplified samples whose absorbance was not readable. We initially used oligos able to anneal at 60°C to amplify the desired fragments. We then optimized MgCl2 concentrations, TAQ polymerase, the oligos/nucleic acid ratio, and the time and cycle number of the amplification step. Figure 1A and 1B show seven different amplification fragments in the *RAS*-*RAF* multiplex and eight distinct bands in the *RET* gene multiplex.

**Figure 2:**
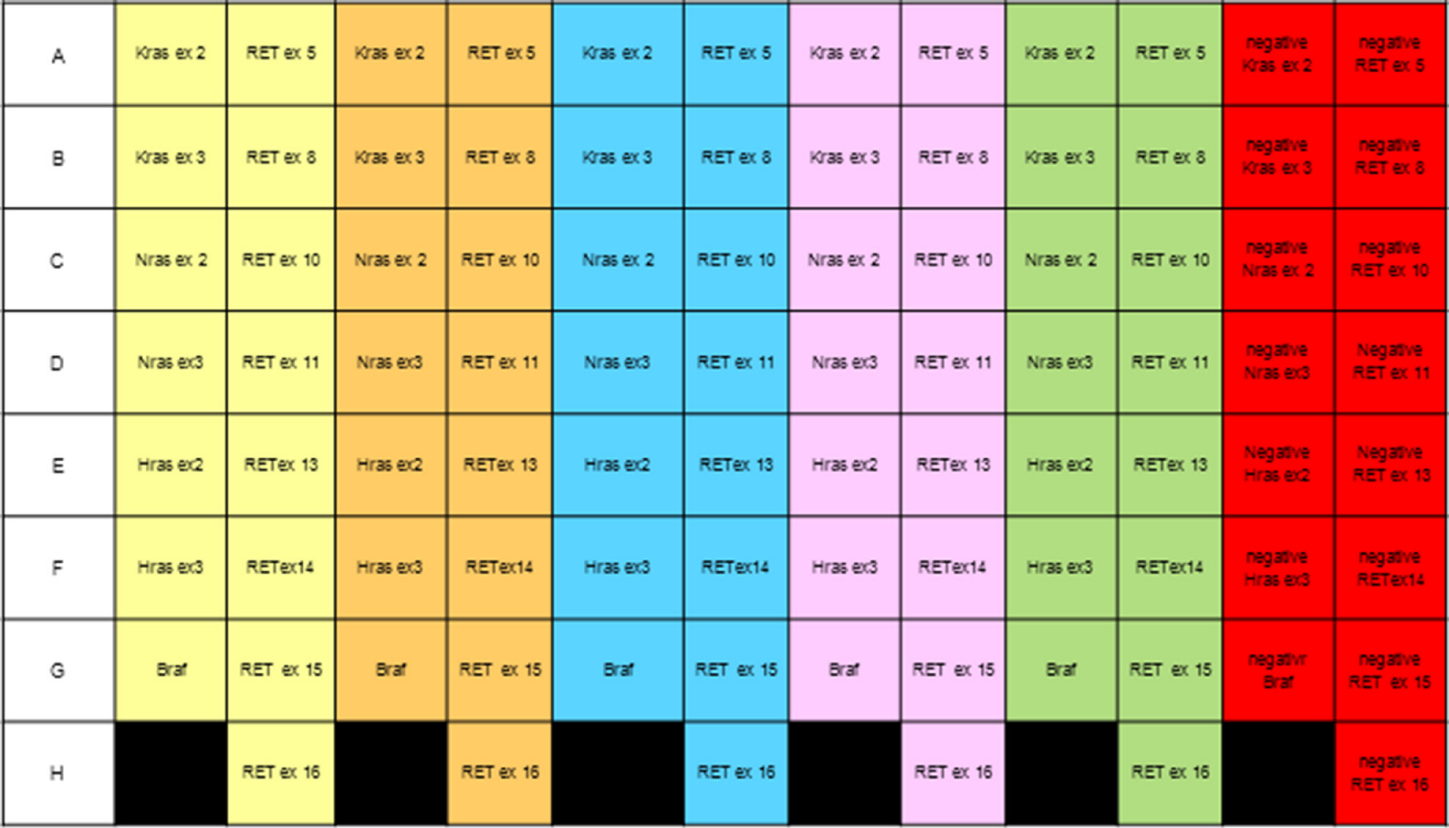
Scheme of a 96-well plate to analyze five samples simultaneously Each color corresponds to a single sample; negative controls are shown in red.

Table 1 shows the results obtained with 15 samples classified according to the “Italian thyroid cytology classification system” [17]. We identified 16 point mutations on 15 samples (one sample contained two mutations). Although the number of samples is not sufficient for statistical validation of the results, it does enable us to verify the performance of our method. We are now collecting more cytological samples to evaluate the diagnostic accuracy of the procedure. We also used FFPE DNA and thanks to the use of a 96-well plate (Figure 2) it was possible to automatically analyze five different samples simultaneously. Consequently, the assay is faster and less expensive than those currently used. Also *RET/PTC1* and *RET/PTC3* amplification was optimized to the 60° annealing temperature, but neither the *RET/PTC1* nor the *RET/PTC3* rearrangement was detected in our samples. Due to the concomitant amplification of gene fragments at the same annealing temperature, the procedure is both rapid and practicable.

**Table 1:** Molecular analysis of 15 FNA thyroid samples

	BRAF	NRAS exon3	RET			
SAMPLES			ex 11 mut691	ex 11 mut769	ex 14 mut836	ex 15 mut904
**TIR2 (2)**			1			
**TIR3 (5)**		1	1	1	1	
**TIR4 (4)**	2	1				
**TIR5 (3)**	1	1	1	1		
**OTHER (1)**	1		1	1		1
**Total 15**	4/15 (27%)	3/15 (20%)	9/15(60%)			
**Mutations/ Samples**	**16/15**					

Fine needle cytology combined with molecular genetics is now the most reliable procedure for the preoperative diagnosis of differentiated thyroid carcinoma [18]. In fact, molecular analysis can provide additional information in multiple histologic variants of well-differentiated PTC. An example is the follicular variant of PTC (FVPTC), which although being a well-defined histopathological entity, its diagnosis on FNA is usually missed. Recently, a new classification of Encapsulated Follicular Variant Papillary Thyroid Carcinoma (eFVPTC) without capsular or vascular invasion in noninvasive follicular thyroid neoplasm with papillary-like nuclear features (NIFTP) was described that is treated more conservatively than classical PTC (cPTC) [19, 20]. Therefore, it is important to distinguish NIFTP and FVPTC from cPTC at the time of FNA. In some cases, thyroid FNAs with PTC-like cytological features, NIFTP/FVPTC can be distinguished from cPTC by a limited number of cytological features. In other cases, the cytological diagnosis of NIFTP/FVPTC can be challenging due to morphological features that overlap with those of non-neoplastic or benign follicular lesions. Since NIFTP/FVPTC were found to be rarely positive for BRAF or RET/PTC mutations, the detection of BRAF, together with RET/PTC, may represent a robust specific test to improve the accuracy of FNA diagnosis of PTC [21].

**Figure 3:**
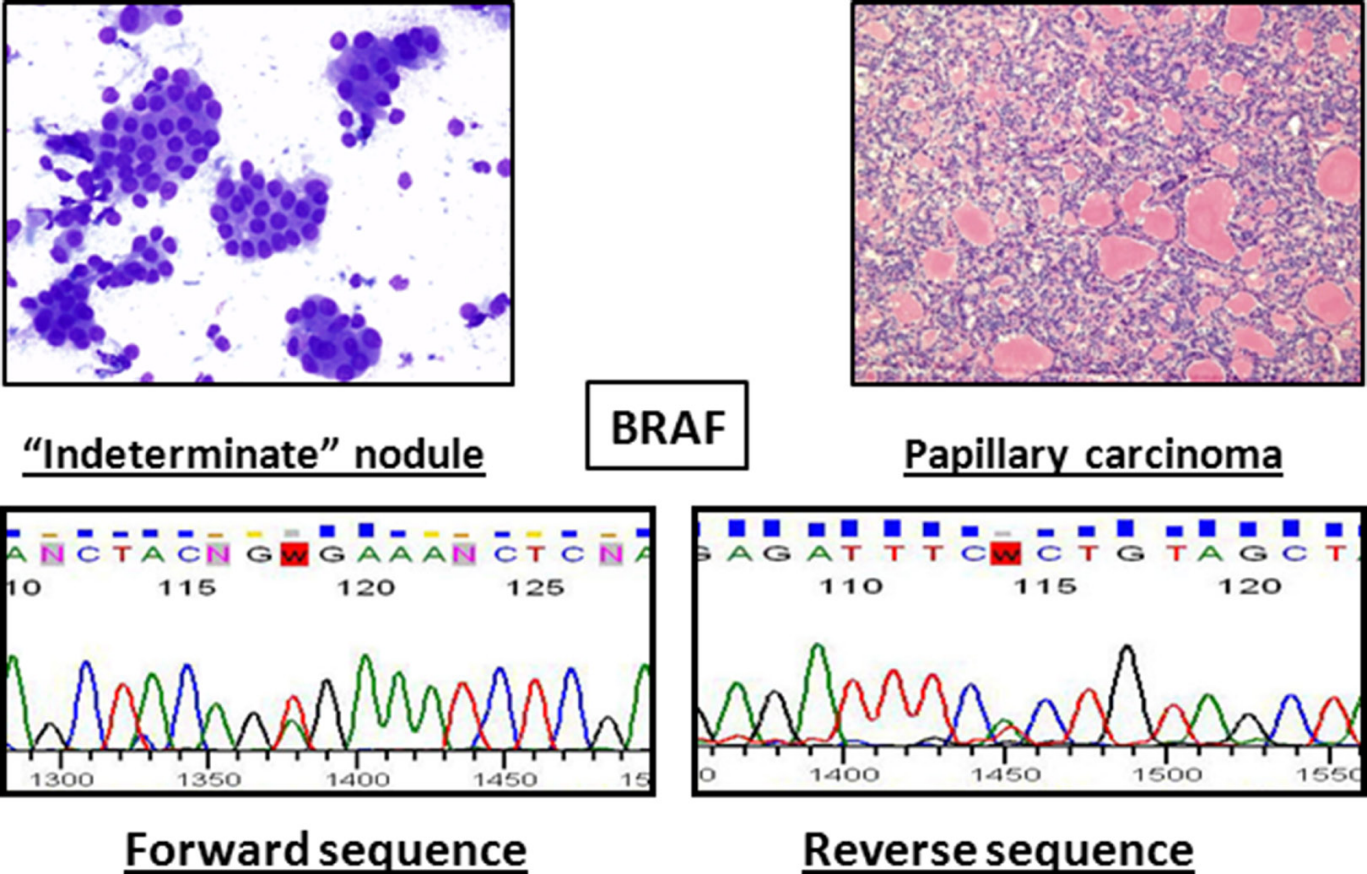
Example of a nodule that was indeterminate by cytology (*left*) and was tested positive for *BRAF* mutation Histological examination revealed a papillary carcinoma (*right*).

The combination of cytology and molecular biology techniques improves the sensitivity and negative predictive value of each approach. Unfortunately, due to the limited number of samples analyzed (15) we are not able to assess the diagnostic accuracy of the molecular markers. Nevertheless, we are collecting a large number of cytological samples to define sensitivity and specificity of each analyzed marker. In any event, it is feasible that the use of a single amplification mix, instead of seven different amplification mixtures, reduces the margin of errors because it involved less sample manipulation. Figure 3 shows an example of an “indeterminate” result obtained by cytomorphology that was resolved by our molecular technique, which revealed a BRAF mutation suggestive of papillary carcinoma.

In conclusion, we describe a new rapid methodology with which to concomitantly analyze several molecular markers of thyroid disease in a single experiment with one reaction for multiplex *BRAF-RAS*, one reaction for eight exons *RET* gene multiplex and two reactions for RNA rearrangement. The methodology can be implemented as a fast screening analysis for the diagnosis and monitoring of cancer patients also in laboratories that do not have access to expensive equipment.

## MATERIALS AND METHODS

### Sampling

Ultrasound-guided fine needle aspiration of thyroid nodules was performed with a 23G gauge needle. The aspirated sample was used for cytology and the residual material and the needle wash were directly injected into a tube containing 1 ml of RLT buffer (Qiagen Germantown, MD, USA) containing 10 μl beta-mercaptoethanol. The tube was stored frozen at −80°C until required for DNA and RNA extraction.

### DNA and RNA extraction

Nucleic acids were extracted with a QIAamp DNA mini kit and an RNeasy mini kit. After thawing a sample, RNA was extracted according to the manufacturer's instructions, but instead of discarding the eluates, these were collected and treated with absolute ethanol and stored overnight at −20°C. The sample was then centrifuged for 30 min. at 10,000 rpm at a low temperature and DNA was extracted using the Qiagen DNA kit procedure. There is a kit for the simultaneous extraction of nucleic acids but we used the separate DNA and RNA extraction Qiagen kit because it is available in our laboratory. The quantity and quality of RNA and DNA were assessed with a NanoDrop 1000 spectrophotomer (Thermo Scientific, Wilmington, DE, USA).

### Mutational analysis

#### BRAF-RAS mutations analysis by PCR multiplex and Ret mutations by PCR multiplex procedures

To set-up our multiplex method, we used a DNA sample from peripheral blood and then we applied our methodology to study 15 FNA samples. The *BRAF, N-H-KRAS* and *RET* exon genes whose mutations were seen to be correlate with disease were detected by amplifications of single gene exons using the c-1000 Thermal Cycler (Bio-Rad, CA, USA). Each exon was amplified in a final volume of 50 μl starting from about 100 ng of gDNA. The amplification mixture consisted of 1X PCR buffer, 200 nmol of dNTPs, 1 pmol/μl of each forward and reverse primer and 2U of Fast Start Taq DNA polymerase (Roche, Pleasanton, CA, USA). In this case, the PCR thermal cycle consisted of initial denaturation at 94°C for 10 min, followed by 35 cycles: denaturation at 95°C for 30 sec, annealing at different temperatures depending on the oligonucleotide used for each region, for 30 sec, and extension at 72°C for 30 sec. Final extension was carried out at 72°C for 10 min.

Then we selected the conditions for each amplification that would enable us to set-up two multiplex PCRs, one to amplify all the exons of *BRAF*, *N-H-KRAS* genes together, and one to amplify eight exons of the *RET* gene. To this aim, we selected primers (Tables 2 and 3) that could all work at the same annealing temperature, and we chose each gene multiplex oligonucleotide not only according to melting temperature (to enable annealing amplification at 60°C) but also based on the lengths of the amplification products, which must be well recognizable fragments. We tested many oligonucleotide pairs for each exon. In detail, we tested 5 and 6 oligonucleotides for *KRAS* exons 2 and 3, respectively, 4 oligonucleotides for *NRAS* exons 2 and 3, 4 oligonucleotides for *BRAF* exon 15, and 16 oligonucleotides for *HRAS* exons 2 and 3. Many single oligonucleotides work well in *HRAS* amplification but not in the multiplex amplification. In addition, long stretches of *HRAS* exons 2 and 3 (> 600 bp) could not be amplified because of poor quality DNA samples. We determined the optimal multiplex concentration for each pair of oligonucleotides based on the results of many individual amplifications. The final concentration of all multiplex amplification primers for the *RET* gene was 1 pmol/μl, while the multiplex *BRAF-RAS* primer concentration was respectively 0.2 pmol/μl for *KRAS*, *NRAS* and *HRAS* exon 2, 0.4 pmol/μl for *BRAF* exon 15 and *HRAS* exon 3, 0.6 pmol/μl for *NRAS* exon 3, and 1.2 pmol/μl for *KRAS* exon 3.

**Table 2:** Primers for RET point mutations

Gene	Sequence 5′ ---> 3′
RET ex5 Forward	TCGCCTGCACTGACCAAC
RET ex5 Reverse	TGTGCATGTGTGTAGGGTGC
RET ex8 Forward	TCCTTGGGCACTAGCTGGA
RET ex8 Reverse	GTTTCCACCGGTGCCATC
RET ex10 Forward	GGGCCTATGCTTGCGACACCA
RET ex10 Reverse	CCAGAGGGAGGGAGGGAAGTTT
RET ex11 Forward	ATACGCAGCCTGTACCCAGT
RET ex11 Reverse	CCTCGTCTGCCCAGCGTTG
RET ex13 Forward	AGAAGCCTCAAGCAGCATCGTC
RET ex13 Reverse	AGGAGCAGTAGGGAAAGGGAGAAA
RET ex14 Forward	TCCTGGAAGACCCAAGCT
RET ex14 Reverse	ATATGCACGCACCTTCATC
RET ex15 Forward	CTGCCATGTCACACCCTG
RET ex15 Reverse	GCTCCACTAATCTTCGGTATCTT
RET ex16 Forward	TCTCCTTTACCCCTCCTTCC
RET ex16 Reverse	TGTAACCTCCACCCCAAGAG

PCR Multiplex 1. Oligonucleotides used for the multiplex detection of the most important mutation regions of the *RET* gene exons: 5 (codon 338), 8 (codons 532, 533), 10 (codons 603, 609, 611, 618 and 620), 11 (codons 630, 631, 634, 635, 637 and 691), 13 (codons 768, 790 and 791), 14 (codons 804, 806, 836 and 844), 15 (codons 883, 891 and 904) and 16 (codons 912).

**Table 3:** Primers for point mutations in the B-RAF, H, K and N-RAS genes

Gene	Sequence 5′ ---> 3′	Work concentration (pmol/μl)
BRAF ex15 Forward	CTCATCCTAACACATTTCAAGCC	0.4
BRAF ex15 Reverse	CTATAGTTGAGACCTTCAATGACTTTC	0.4
HRAS ex2 Forward	TGGCTGAGCAGGGCCCTCCT	0.2
HRAS ex2 Reverse	CTGCTGGCACCTGGACGGCGGC	0.2
HRAS ex3 Forward	GGCATGAGAGGTACCAGGGAGA	0.4
HRAS ex3 Reverse	AGGACAGGAGGCCCCTGCCTGGAC	0.4
KRAS ex2 Forward	GGTACTGGTGGAGTATTTGATAGTG	0.2
KRAS ex2 Reverse	CTGACATACTCCCAAGGAAAGTAAAG	0.2
KRAS ex3 Forward	TCCCTTCTCAGGATTCCTACAGG	1.2
KRAS ex3 Reverse	CCCACCTATAATGGTGAATATC	1.2
NRAS ex2 Forward	AGAACCAAATGGAAGGTCAC	0.2
NRAS ex2 Reverse	GTGAGAGACAGGATCAGGTC	0.2
NRAS ex3 Forward	TGAGGGACAAACCAGATAGGC	0.6
NRAS ex3 Reverse	CTGTAGAGGTTAATATCCGCAAATG	0.6

PCR multiplex 2: Oligonucleotides used for the multiplex detection of exon 15 of the *BRAF* gene, point mutations in codons 12, 13 and 61 (hotspots) of the *HRAS* gene and in codons 12, 13, 59, 61 of *K-NRAS* genes. The concentration primers in the mix is reported.

We tested five different Taq DNA polymerases that are available in our laboratory: HotMaster Taq DNA Polymerase (5 Prime, Gaithersburg, USA); AmpliTaq Gold (Thermo Fisher Scientific, Waltham, MA, USA); FastStartTaq DNA Polymerase, Expand High Fidelity PCR System and Taq DNA Polymerase (Roche), and found the best working conditions with FastStartTaq Polymerase. FastStartTaq DNA Polymerase is a thermostable, modified form of recombinant Taq DNA polymerase. It is inactive at temperatures below 75°C, but can be activated by a 4 min 95°C incubation step. The combination of FastStartTaq DNA Polymerase and the dedicated PCR buffer minimizes non-specific amplification products and primer dimers. In most cases, the system easily amplifies multiple templates in a single reaction.

We also tested different concentrations of MgCl_2_ and found that a standard concentration 1.5 nM was optimal. We also changed the multiplex temperature profile from that of single amplifications to enable many oligos to act simultaneously; in particular, annealing time was prolonged from 30 sec to 1 min 30 sec, elongation time from 30 sec to 1 min, and the cycles were decreased from 35 to 30.

Fine needle cytology generally results in small samples, thus we tested the smallest amount that enabled good amplification in multiplex. We used 50, 100 and 200 ng and the best amplification was obtained with 100 ng of DNA for each amplification multiplex PCR, for a total of 200 ng. This is a very small amount of DNA compared with the 1,500 ng required for all single amplifications. We also used formalin-fixed paraffin-embedded (FFPE) DNA extracted with the QIAamp@DNA FFPE tissue kit (Qiagen) according to the supplier's recommendations to verify that our multiplex PCR worked also with nucleic acids obtained from FFPE present in archival material. In this case, more DNA was required (the optimum was about 300 ng). Using FFPE DNA in a 96-well plate with the thermal profiles reported for the individual amplifications, we were able to automatically analyze five different samples simultaneously. Thus, the procedure can be automated and all stages (amplification reaction, post-amplification purification sequence amplification, post-sequencing purification, dilution and sample preparation for capillary electrophoresis) can be performed on a liquid handler (Beckman Coulter BFX).

An oligonucleotide design strategy using primers bound in reverse and forward to the universal M13 sequences (M13 Fw: CGTTGTAAAACGACGGCCAGT; M13 Rev: TTTCACACAGGAAACAGCTATGAC) can simplify the method further.

All amplifications were gel controlled (2% agarose gel in 1X TBE) using molecular weight marker (Fermentas Mass Ruler 100 bp ladder) with the negative controls to verify successful amplifications. Amplicon lengths ranged between 195 and 447 bp in the *BRAF-RAS* PCR multiplex and between 176 and 447 bp in the *RET* PCR multiplex. All positive PCR products were purified using the High Pure PCR Product Purification Kit (Roche) or the Gen Elute Gel Extraction Kit (Sigma-Aldrich St. Louis USA), and diluted 1:8 to optimize the sequencing conditions. Two μl diluted samples were used in the next sequence reaction mix and sequenced on both strands (Life Technologies Big dye Terminator v3.1 Chemistry) by capillary electrophoresis performed with the Life Technologies 3730 DNA Analyzer. All sequencing reactions were performed with the same oligonucleotides used for the amplifications, except in the case of the *HRAS* gene in which more internal primers were used (see Table 4).

**Table 4:** Primers for the sequences of HRAS exons 2 and 3

Gene	Sequence 5′ ---> 3′
HRAS ex2 seqForward	CTGTAGGAGGACCCCG
HRAS ex2 seqReverse	CGCCAGGCTCACCTCTAT
HRAS ex3 seqForward	ATTCCTACCGGAAGCAGGTGG
HRAS ex3 seqReverse	CTCACGGGGTTCACCTGTACTG

### RET-PTC rearrangements

0.5 μg of RNA was reverse transcribed into cDNA as indicated in the Qiagen “QuantiTect reverse transcription kit”. The cDNA was stored at −20°C until further use. The housekeeping gene GAPDH, which is uniformly expressed in all cells, was amplified by PCR to verify that the quality of the RNA samples was suitable for molecular analysis. *RET*-PTC rearrangements 1 and 3 were detected by PCR amplification on the c-1000 Thermal Cycler (Bio-Rad) and primer sequences (GAPDH included) are reported in Table 5. The cDNAs from the TPC-1 cell line and a PTC3 positive sample (previously typed) served as controls. PCR was performed in total volume of 25 μl using 5 μl of cDNA. The amplification mixture consisted of 1X PCR buffer (Roche), 1 pmol/μl of forward and reverse primers, 200 nmol of dNTPs and 1U of Taq DNA polymerase (Roche). All amplifications were gel controlled (2% agarose gel in 1X TBE) but only positive PCR products were purified and analyzed by direct sequencing.

**Table 5:** Primers for the housekeeping gene GAPDH, and for RET/PTC1 and RET/PTC3 gene rearrangements

Gene	Sequence 5′ ---> 3′
RET/PTC1 Forward	ATTGTCATCTCGCCGTTC
RET/PTC1 Reverse	CTTTCAGCATCTTCACGG
RET/PTC3 Forward	TGGAGAAGAGAGGCTGTATC
RET/PTC3 Reverse	CGTTGCCTTGACCACTTTTC
GAPDH Forward	CCCTTCATTGACCTCAACTACATG
GAPDH Reverse	TGGGATTTCCATTGATGACAAGC
